# Cheap Benevolence

**Published:** 1891-10-24

**Authors:** 


### PASSING TOPICS.

### CHEAP BENEVOLENCE.

It has been truly said that many people have feelings but no heart, and similarly we may predicate of the sympathies of some, and an increasing number, that though easily called into existence, they have little of the vitality which alone renders them productive. Like certain plants, they seem to spring into being the more readily when the soil is poor, and having no strength to carry them to maturity, they wither away after a brief and unfruitful life.

But sympathy and feelings exist in different degrees, and not only must they operate upon diverse temperaments, but of necessity they depend upon various conditions. There are the men and women whose compassion for want and suffering is genuine enough, but who are too busy or too poor to take any active part in its relief; there are others—all too few—who possess alike the will and the power, and there is a third class of persons who possess the power and boast the sympathy, but are yet content to state the case and to call upon their neighbours to assist. These are the people occasionally of high rank, and, more often than not, of great wealth, who write to the papers inviting subscriptions for all kinds of objects more or less worthy and more or less microscopical. A lady—it is usually a lady—of position thinks nothing nowadays of sending a letter to the *Times* or the *Post*, asking for public help to raise a sum which represents little more than the price of a new bonnet or a box at the theatre. Nothing is too trivial to be utilised in this way. A gift of pathos and a power of invocation are as lavishly displayed over the misfortunes of an individual as in the history of a great and far-reaching catastrophe, and the vocabulary is emptied alike for a description of a direful accident to the village postman's wooden leg and the narrative of the unexpected and inconvenient arrival of triplets in a roadside cottage. Some local magnate, we are obligingly informed, will gladly receive subscriptions. Of course he will if he gets any, but his willingness to acknowledge and apply them is, alas! not so often put to the test, and we fear the result is seldom anything but disappointment to the person chiefly concerned, who has naturally hoped for much.

What is it which urges rich people thus to accentuate the barrenness and impotence of their abundant sympathies? "Here," they cry, "is distress; do you, good reader, relieve it while I pass by, and on the other side of the street reveal a fresh tale of suffering." This ability to discover objects which other people may advantageously espouse, is a truly artistic gift, but it may be questioned whether three lines on a cheque would not be more efficacious than a ream of advocacy. The reader's sense of compassion is more often than not lost in one of irritation. In these captious times, a display of amiability is not always received with gratification. Little is taken upon trust, and when people notice these effusions they are very apt to become cynics for the nonce, and to inquire, unreasonably of course, what can be the real motive for inviting all England to assist at so very small a function. AGRICOLA.

